# Adolescent Problem Gambling in Rural Ghana: Prevalence and Gender Differentiation

**DOI:** 10.1007/s10899-020-09987-6

**Published:** 2020-11-11

**Authors:** Samuel Kofi Odame, Emmanuel Nii-Boye Quarshie, Mabel Oti-Boadi, Johnny Andoh-Arthur, Kwaku Oppong Asante

**Affiliations:** 1 Department of Sociology, University of Ghana, Accra, Ghana; 2 School of Psychology, University of Leeds, Lifton Place, Leeds, LS2 9JT West Yorkshire UK; 3 Department of Psychology, University of Ghana, Legon, Accra, Ghana

**Keywords:** Adolescents, Gambling, Ghana, Problem gambling, Rural adolescents

## Abstract

Problem gambling among young people is now a public health challenge in sub-Saharan Africa. However, the behaviour remains understudied, particularly, among rural-dwelling young people in countries within the subregion. We aimed to estimate the 12 months prevalence of problem gambling and to describe the overall and gender differences and commonalities in personal factors and social adversities associated with problem gambling among adolescents in rural Ghana. We conducted a cross-sectional survey involving a random sample of 1101 in-school adolescents aged 10–19 years in a rural district in Eastern Ghana; we used the DSM-IV-Multiple Response-Juvenile (DSM-IV-MR-J) questionnaire to assess problem gambling during the previous 12 months. Personal lifestyle and psychosocial variables were assessed using adopted items from the 2012 WHO–Global School-based Student Health Survey. Overall, three in 10 adolescents (3 in 10 females; 4 in 10 males) in rural Ghana reported problem gambling in the previous 12 months. Female adolescents who experienced problem gambling were more likely to report family-related social adversities, while adolescent male problem gambling was associated with school-related factors and interpersonal factors outside the family context. Regardless of gender, sexual abuse victimisation was associated with three times increase in the odds of experiencing problem gambling. Relative to the prevalence of gambling among adolescents in urban contexts in other countries within sub-Saharan Africa, the estimates of problem gambling among in-school rural adolescents in Ghana are higher. Although further studies are needed to understand the nuances of the behaviour, the evidence of this study underscores the need for general and targeted health promotion, intervention and prevention efforts to mitigate the family, school, and interpersonal social adversities associated with adolescent problem gambling in rural Ghana.

**Electronic supplementary material:**

The online version of this article (10.1007/s10899-020-09987-6) contains supplementary material, which is available to authorized users.

## Introduction

Gambling among young people has become an important public health concern (Derevensky et al. 2011; Messerlian et al. 2005). Problem gambling among young people has been attributed to the emergence and development of technology–with new gambling forms via mobile phones, television and the Internet (Griffiths & Parke, 2010; Messerlian et al. 2004; Monaghan et al. 2008). Availability and easy access appear to underpin the appeal of these new forms of gambling to young people (Delfabbro et al. 2009). Problem gambling refers to gambling activities that lead to a continuous or periodic loss of control over gambling that is marked by erroneous cognitions, irrational thinking, continued gambling in spite of severe negative consequences, a preoccupation with gambling and obtaining money to gamble, and an inability to stop gambling (American Psychiatric Association 1994).

General theories of human behaviour such as psychoanalysis and social learning have also been used to explain gambling. Within the psychoanalysis perspective, gambling is viewed in terms of erotisation of fear or a sublimation of oedipal aggression toward the father (Aasved 2002; Lindner 1950). The gambler’s characteristics are “derived from attempts to obtain, through the mechanism of reverting to earlier infantile ways of conduct, the ‘narcissistic supplies’ —i.e., food, love, comfort, and attention” which the gambler believes they have been denied (Lindner 1950). The social learning perspective suggests that gambling is actively undertaken ‘socially' by the majority of people in society and even ‘addiction' is couched in social processes; children of parents with gambling problems are also likely to develop gambling problems through observation (Binde 2013).

Some empirical studies have identified key gambling motivations, ranging from financial rewards to non-financial outcomes such as amusement, escape, wining, and sensation seeking (Binde 2013; Lee et al. 2006; McGrath et al. 2010; Neighbors et al. 2002). A longitudinal qualitative study has suggested that social processes within significant social networks rather than purely individual characteristics or deviant motivations underlie young peoples’ index gambling behaviours (Kristiansen et al. 2015).

Globally, about 12.3% of young persons have been found to show problem gambling behaviour (Calado et al. 2017; Dowling et al. 2017; Sapthiang et al. 2020). For instance, a cross-national study from Europe suggests that about 1.6%–5.3% of adolescents report probable problem gambling (Molinaro et al. 2014). Male-females estimates of severe gambling-related problems among adolescents have been found to range between 3:1 and 5:1, inadvertently leading to a disproportionate focus on male gambling behaviours in the gambling literature (Ellenbogen et al. 2007; Jacobs 2004). Although some recent studies have focused exclusively on female gambling behaviours (Derevensky et al. 2011; Derevensky and Gupta 2005a; Huic et al. 2017), it is worthy to note that for both males and females with severe gambling problems, strong comorbidity with depression and substance use, and other risk behaviours have been found (Ellenbogen et al. 2007).

Several recent systematic reviews and meta-analyses of the literature (mainly from high-income countries) have reported some key risks and correlates of problem gambling among young people, including being male, younger, psychological distress, substance use, child maltreatment, parental problem gambling (Dickson et al. 2008; Dowling et al. 2017; Griffiths and Parke 2010; Lane et al. 2016; Nowak 2018; Shead et al. 2011). Generally, even though gambling may provide some psychosocial benefits (Binde 2013), comparatively, problem gambling has been associated with various negative outcomes, including mental and physical health problems, relationship and family dysfunction, financial problems, employment difficulties, and legal issues (Blinn-Pike et al., 2010; Derevensky et al. 2011; Derevensky and Gupta 2005a; Langhinrichsen-Rohling 2005; Shaffer and Hall 2002).

## Adolescent Gambling in Sub-Saharan Africa

Little is known about gambling in sub-Saharan Africa, while problem gambling among young people in countries within the subregion has received little research attention (Ssewanyana and Bitanihirwe, 2018).^1^ A recent rapid survey found that 54% of youth in sub-Saharan Africa have engaged in some form of gambling activity (GeoPoll 2017). Kenya topped the list of countries with higher prevalence of gambling behaviour among the young persons at 76%; Uganda followed with 57%, and 42% from Ghana (GeoPoll 2017). Evidence from Ethiopia shows a prevalence estimate of 73% among high school adolescents, with about 37% of these adolescents being at risk of severe problem gambling (Abdi et al. 2015). An urban school-based cross-sectional survey involving 507 students in Nigeria also found a lifetime gambling prevalence of 57.2%, out of which 77.6% had gambled in the previous 12 months (Aguocha et al. 2019). Generally, self-esteem, false perceptions about winning, drug abuse, peer influence, parental gambling, and the accessibility of gambling venues are significantly associated with problematic gambling within sub-Saharan Africa (Abdi et al. 2015; Aguocha et al. 2019; Glozah et al. 2019; Tagoe et al. 2018).

## Adolescent Gambling in Ghana

Underage gambling is criminalised in Ghana (Gaming Act of Ghana 2006); however, emerging studies suggest that underage gambling is a reality in both urban and rural communities in the country (Glozah et al. 2019; Hayk and Sailer 2020; Tagoe et al. 2018; Tolchard et al. 2014). Major gambling activities among young people range from sport betting to lotteries: sports betting (21.1%), card games (4.5%), poker machines (2.9%), and lotteries (1.7%) (Glozah et al. 2019). Notably, these gambling activities are popular in urban areas, while Chinese slot machines are popular in rural communities in Ghana (Hayk and Sailer 2020). However, the regulation of the activities of gambling centres and operators of gambling machines in rural communities remains a critical challenge–thereby creating cosmopolitan encounters in the rural communities, while potentially threatening the psychosocial health status of rural young people (Hayk and Sailer 2020).

Thus far, only two studies are available from urban Ghana with specific focus on adolescent gambling (Glozah et al. 2019; Tagoe et al. 2018), with no study providing evidence on the prevalence and potential correlates of problem gambling among rural adolescents–although recent evidence suggests that rural adolescents patronise slot machines (Hayk and Sailer 2020). This study has two aims: (1) to estimate the 12 month prevalence of problem gambling among adolescents^2^ in rural Ghana, and (2) to identify the overall, and gender differences and commonalities in personal factors and social adversities associated with problem gambling among adolescents in rural Ghana.

## Methods

In designing and conducting the current study and writing this paper, we have been guided by the Strengthening the Reporting of Observational Studies in Epidemiology (STROBE) recommendations for cross-sectional study (Von Elm et al. 2007).

### Design, Setting, and Participants

We conducted a cross-sectional survey, using a self-report questionnaire, among regularly attending Junior High Schools^3^ (JHS) adolescents in the Ayensuano rural district in the eastern region of Ghana. A cross-sectional survey is appropriate for the retrospective estimation of the prevalence an outcome variable, and for the identification of the correlates of an outcome (Woodward 2014). We obtained the list of schools and pupil statistics from the District Educational Directorate of Ayensuano: there were 84 JHS being attended by 9365 students. Based on an estimated prevalence of 42% of mobile gambling among young people in Ghana (GeoPoll 2017), we predetermined a minimum sample size of 971 students, using Cochran’s formula for calculating a sample for proportions (Cochran 1963). However, the final predetermined sample (n = 1214) included an additional 25% for two reasons: to obtain satisfactory precision and confidence interval widths, and to compensate for nonresponse (Naing et al. 2006). In all, we randomly selected and approached 15 schools proportional to enrollment size to participate in the study; however, the heads of 12 schools permitted the study in their schools (representing a school response of 80%). Across the 12 selected participating schools, we randomly selected 36 classes proportional to enrollment size. In each selected class, all the students qualified to respond to the survey–we invited all the students in a selected class to participate in the survey. Students who were willing to respond to the survey and provided their consent were included, whereas students who were absent on the day of the survey were excluded from the study. In all, we approached and invited 1214 students to respond to the survey, but, 1101 adolescents (female = 575; male = 526) provided complete data included in the study–representing a response of rate of 90%.

## Measures

The participants responded to a self-report anonymous questionnaire composed of the following sub-sections: socio-demographic variables, problem gambling, personal factors and social adversity.

### Socio-Demographic Variables

We included seven items to assess the social characteristics and demographic background of the participants: gender (female or male), age, grade, living arrangement, caretaker’s employment status, family structure (measured by father’s number of wives), and romantic relationship status (see Table 1).

**Table 1:** Demographics

Variable		Total sample	Adolescent group		Difference in proportions (95% CI for difference)
		(N = 1101)	Females (n = 575)	Males (n = 526)	
		n (%)	n (%)	n (%)	
Age	Younger adolescents (10–15 years)	516 (46.9)	294 (51.1)	222 (42.2)	0.089 (0.03, 0.15)
	Older adolescents (16–19 years)	585 (53.1)	281 (48.9)	304 (57.8)	− 0.089 (− 0.15, − 0.03)
Grade	JHS 1	708 (64.3)	389 (67.7)	319 (60.6)	0.071 (0.01, 0.13)
	JHS 2	279 (25.3)	127 (22.1)	152 (28.9)	− 0.068 (− 0.12, − 0.02)
	JHS 3	114 (10.4)	59 (10.3)	55 (10.5)	− 0.002 (− 0.04, 0.03)
Living arrangement	Live with both parents	606 (55)	321 (55.8)	285 (54.2)	0.016 (− 0.04, 0.07)
	Live with one parent	336 (30.5)	172 (29.9)	164 (31.2)	− 0.013 (− 0.07, 0.04)
	Live with no parents	159 (14.4)	82 (14.3)	77 (14.6)	− 0.003 (− 0.04, 0.04)
Caretaker's employment status	Unemployed	116 (10.5)	45 (7.8)	71 (13.5)	− 0.057 (− 0.09, − 0.02)
	Employed	985 (89.5)	530 (92.2)	455 (86.5)	0.057 (0.02, 0.09)
Family structure	Father has 1 wife	702 (63.8)	378 (65.7)	324 (61.6)	0.041 (− 0.01, 0.09)
	Father has > 1 wife	399 (36.2)	197 (34.3)	202 (38.4)	− 0.041 (− 0.09, 0.02)
In romantic relationship	No	632 (57.4)	305 (53)	327 (62.2)	− 0.092 (− 0.15, − 0.03)
	Yes	469 (42.6)	270 (74)	199 (37.8)	0.362 (0.03, 0.15)

### Problem gambling

We used the Diagnostic and Statistical Manual of Mental Health Fourth Edition Adapted for Juveniles (DSM-IV-MR-J) questionnaire (Fisher 2000) to assess problem gambling during the previous 12 months. The DSM-IV-J is a 12 item checklist which assesses nine criteria of problem gambling: progression and preoccupation, tolerance, withdrawal and loss of control, escape, chasing, lies and deception, illegal activities, family and school disruptions, and financial bailout (Jacques and Ladouceur 2003). Predominantly, the items are on a 4 point rated scale: never, once or twice, sometimes, and often. The presence of four (or more) out of the nine criteria indicates problem gambling (Fisher 2000; Jacques and Ladouceur 2003). Example, *In the past year have you ever spent much more than you planned to on gambling?* Answers are transformed into dichotomous responses (presence–absence of problem gambling, based on the criteria).The DSM-IV-J scale has an acceptable internal consistency (Cronbach’s alpha = 0.75) (Fisher 2000) and has been used extensively across the world (Derevensky and Gupta 2005b; Gupta and Derevensky 2000). In sub-Saharan Africa, the scale has demonstrated satisfactory validity and reliability (Abdi et al. 2015; Ssewanyana et al. 2018). The Cronbach’s alpha score in the current study was 0.81.

### Personal Factors and Social Adversity

We included eight binary (No or Yes) response rated personal lifestyle factors and variables assessing the experience of interpersonal adversities, adopted from the 2012 WHO–Global School-based Student Health Survey questionnaire used in Ghana (Owusu 2012). These variables included: *weekly alcohol use* (In a typical week, how many times do you have at least on alcoholic drink?), *truancy* (During the past 12 months, how many days did you miss classes or school without permission?), *bullying victimisation* (During the past 12 months, how many days were you bullied?), and *sexual abuse victimisation* (Has anyone forced you [i.e. physically or verbally] to engage in sexual activities against your will?). We also adopted one item from the 5 item Duke University Religion Index (DUREL) (Koenig and Büssing 2010) to assess *religious participation*: “How often do you attend church or other religious meetings?” with response rating options ranging from (1) ‘never’ to (6) ‘more than once per week’. We have shown in the supplementary material the variables included in this study and the specific questions used to assess them in the survey (eTable 1).

## Procedure

The administration of the survey took place between August 2019 and January 2020. The participants of the study were gathered in their school’s assembly hall or a larger classroom designated for the survey, with sitting arrangement spaced by reasonable distance. After obtaining permission from the heads of the participating schools, informed consent of parents/guardians, and the participants’ informed consent and assent, we gave each student a packet of the anonymous questionnaire to answer. Averagely, the completion of the questionnaire lasted between 22 and 35 min. Upon completing the survey, each participant placed their answered questionnaire in an opaque box placed near the exit door.

### Data Analysis

The Statistical Package for Social Sciences (SPSS version 26.0 for Windows) was used for the analysis. Gambling (non-problem gambling vs. problem gambling) was the outcome variable, while the exposure variables or correlates were composed of the socio-demographic variables, personal factors and the variables of social adversity specified. As the loss of cases due to missing data was less than 5%, we used the list‐wise deletion of missing data strategy (Graham 2009). We have shown in the supplementary material the coding of the variables included in our statistical analysis (eTable 1). The data analysis proceeded in three stages; at each stage, we stratified the data by gender (female and male), guided by the aims of the study. Firstly, we performed descriptive analysis of the data by applying frequencies, proportions and the frequentist 95% confidence intervals (CI) (Hespanhol et al. 2019) to assess the uncertainty around the effect estimates of the difference in proportions (DP) between female and male adolescents in terms of social demographic variables and the 12-month prevalence estimates of problem gambling. In stage two, given the categorical nature of the data, we applied the Pearson’s Chi-squared test (${\upchi })$ to explore the bivariate relationships between problem gambling and each of the socio-demographic variables, personal factors and the variables of social adversity included in the study. We performed a point-biserial correlation (*r*_pb_) test to examine the possible bivariate relationship between religious participation and problem gambling (Prematunga 2012). Statistically significant results were determined using the *p *value less than 0.05 (*p* < 0.05).The final stage of the analysis involved multivariable logistic regression, to examine the possible associations between the binary outcome variable (gambling: non-problem gambling vs. problem gambling) and the specified correlates (socio-demographic variables, personal factors, and variables of social adversity). We built three models, one each for the overall sample, female adolescent sub-sample, and the male adolescent sub-sample. As recommended by leading logistic regression modelling experts, the candidate correlates were entered in the multivariable logistic regression models regardless of the statistical significance of their bivariate relationship with the outcome variable (Babyak 2004). We reported the results of the logistic regression as odds ratios with 95% confidence intervals (CI) and *p* values (Greenland et al. 2016).

## Results

### Sample Characteristics

The 1101 participants in this study were aged 10–19 years (mean = 15.3; modal = 16; SD = 1.9). There were more female (n = 575) than male (n = 526) adolescents. Table 1 presents the socio-demographic characteristics of the participants, stratified by gender.

While the majority of younger adolescents were females (n = 294 [DP 0.089, 95% CI 0.03, 0.15]), most of the older adolescents were males (n = 304 [DP − 0.089, 95% CI − 0.15, − 0.03]). The majority of the participants were in JHS 1 (64.3%), lived with both parents (55%), and identified their family as monogamous (63.8%). More females (n = 270) than males (n = 199) indicated as being in a romantic relationship (DP 0.36, 95% CI 0.03, 0.15).

### Prevalence Estimates of Problem Gambling

Overall, 378 (34.3%) participants reported problem gambling during the previous 12 months. Regarding gender, more male adolescents (n = 204; 38.8%) than females (n = 174; 30.3%) reported problem gambling during the previous 12 months [DP 0.08, 95% CI 0.029, 0.141]. In terms of age, more younger adolescents (n = 200; 38.8%) reported problem gambling during the previous 12 months, compared to older adolescents (n = 178; 30.4%) [DP 0.08, 95% CI 0.027, 0.14]. The prevalence estimates of problem gambling were varied across grade: JHS 1 (n = 242; 34.2%), JHS 2 (n = 108; 38.7%), and JHS 3 (n = 28; 24.6%).

### Bivariate Associations

Across the overall sample and gender, we found statistically significant bivariate associations between problem gambling and most of the socio-demographic variables, personal factors and social adversities included in this study (see Table 2).

**Table 2:** Bivariate analysis of socio-demographic variables, personal factors and social adversities associated with problem gambling in the previous 12 months, stratified by gender

Variable	Total sample				Female				Male			
	Problem gambling in the past 12 months				Problem gambling in the past 12 months				Problem gambling in the past 12 months			
	No n (%)	Yes n (%)	${\upchi }$	*p *value	No n (%)	Yes n (%)	${\upchi }$	*p* value	No n (%)	Yes n (%)	${\upchi }$	*p *value
***Demographic variables***												
Gender			8.85	.003								
Female	401 (96.7)	174 (30.3)										
Male	322 (61.2)	204 (38.8)										
Age			8.44	.004			4.77	.029			5.46	.019
Younger adolescents (10–15 years)	316 (61.2)	200 (38.8)			193 (65.6)	101 (34.4)			123 (55.4)	99 (44.6)		
Older adolescents (16–19 years)	407 (69.6)	178 (30.4)			208 (74.0)	73 (26.0)			199 (65.5)	105 (34.5)		
Grade			7.21	.027			5.38	0.68			1.89	.388
JHS 1	466 (65.8)	242 (34.2)			271 (69.7)	118 (30.3)			195 (61.1)	124 (38.9)		
JHS 2	171 (61.3)	108 (38.7)			82 (64.6)	45 (35.4)			89 (58.6)	63 (41.4)		
JHS 3	86 (75.4)	28 (24.6)			48 (81.4)	11 (18.6)			38 (69.1)	17 (30.9)		
Living arrangement			5.55	.062			1.58	.454			5.35	.069
Live with both parents	414 (68.3)	192 (31.7)			227 (70.7)	94 (29.3)			187 (65.6)	98 (34.4)		
Live with one parent	204 (60.7)	132 (39.3)			114 (66.3)	58 (33.7)			90 (54.9)	74 (45.1)		
Live with no parents	105 (66.0)	54 (34.0)			60 (73.2)	22 (26.8)			45 (58.4)	32 (41.6)		
Caretaker's employment status			2.20	.138			0.017	.897			2.05	.152
Unemployed	69 (59.5)	47 (40.5)			31 (68.9)	14 (31.1)			38 (53.5)	33 (46.5)		
Employed	654 (66.4)	331 (33.6)			370 (69.8)	160 (30.2)			284 (62.4)	171 (37.6)		
Family structure			29.33	.000			15.21	.000			13.08	.000
Father has 1 wife	502 (71.5)	200 (28.5)			284 (75.1)	94 (24.9)			218 (67.3)	106 (32.7)		
Father has > 1 wife	221 (55.4)	178 (44.6)			117 (59.4)	80 (40.6)			104 (51.5)	98 (48.5)		
In romantic relationship			61.27	.000			26.49	.000			41.28	.000
No	476 (75.3)	156 (24.7)			241 (79.0)	64 (21.0)			235 (71.9)	92 (28.1)		
Yes	247 (52.7)	222 (47.3)			160 (59.3)	110 (40.7)			87 (43.7)	112 (56.3)		
***Personal factors***												
Weekly alcohol use			72.02	.000			30.09	.000			41.17	.000
No	616 (72.2)	237 (27.8)			340 (75.2)	112 (24.8)			276 (68.8)	125 (31.2)		
Yes	107 (46.1)	141 (56.9)			61 (49.6)	62 (50.4)			46 (36.8)	79 (63.2)		
Truancy			13.95	.000			3.31	.069			9.36	.002
≤ 5 days	669 (67.4)	323 (32.6)			375 (70.8)	155 (29.2)			294 (63.6)	168 (36.4)		
> 5 days	54 (49.5)	55 (50.5)			26 (57.8)	19 (42.2)			28 (43.8)	36 (56.3)		
Schoolwork problems			37.55	.000			13.77	.000			26.35	.000
No	347 (76.1)	109 (23.9)			179 (78.5)	49 (21.5)			168 (73.7)	60 (26.3)		
Yes	376 (58.3)	269 (41.7)			222 (64.0)	125 (36.0)			154 (51.7)	144 (48.3)		
***Social adversities***												
Bullying victimisation			50.85	.000			23.09	.000			25.51	.000
No	484 (74.1)	169 (25.9)			276 (76.9)	83 (23.1)			208 (70.7)	86 (29.3)		
Yes	239 (53.3)	209 (46.7)			125 (57.9)	91 (42.1)			114 (49.1)	118 (50.9)		
Breakup			73.47	.000			51.75	.000			24.88	.000
No	553 (74.1)	193 (25.9)			309 (79.2)	81 (20.8)			244 (68.5)	112 (31.5)		
Yes	170 (47.9)	185 (52.1)			92 (49.7)	93 (50.3)			78 (45.9)	92 (54.1)		
Sexual abuse victimisation			119.06	.000			83.27	.000			48.84	.000
No	575 (76.3)	179 (23.7)			301 (83.1)	61 (16.9)			274 (69.9)	118 (30.1)		
Yes	148 (42.7)	199 (57.3)			100 (46.9)	113 (53.1)			48 (35.8)	86 (64.2)		
Conflict with parents			50.32	.000			26.64	.000			24.36	.000
No	586 (71.6)	232 (28.4)			322 (75.6)	104 (24.4)			264 (67.3)	128 (32.7)		
Yes	137 (48.4)	146 (51.6)			79 (53.0)	70 (47.0)			58 (43.3)	76 (56.7)		
Parental divorce			41.52	.000			33.71	.000			11.93	.001
No	506 (72.7)	190 (27.3)			280 (78.4)	77 (21.6)			226 (66.7)	113 (33.3)		
Yes	217 (53.6)	188 (46.4)			121 (55.5)	97 (44.5)			96 (51.3)	91 (48.7)		

$${\upchi }^2$$ Chi square

Males were more likely to report problem gambling than female adolescents (${\upchi }^{2}$_(1)_ = 8.85, *p* = 0.003). Comparatively, four variables showed the strongest statistical bivariate correlation with problem gambling across the total sample and gender: sexual abuse victimisation (${\upchi }^{2}$_(1)_ = 119.0, *p* < 0.001), breakup (${\upchi }^{2}$_(1)_ = 73.4, *p* < 0.001), weekly alcohol use (${\upchi }^{2}$_(1)_ = 72.0, *p* < 0.001), and being in a romantic relationship (${\upchi }^{2}$_(1)_ = 61.2, *p* < 0.001).

Furthermore, across the overall sample, there was a statistically significant negative correlation between religious participation and problem gambling during the previous 12 months (*r*_pb_ = − 0.74, *N* = 1090, *p* = 0.015); however, when stratified by gender, there was a statistically non-significant negative correlation between religious participation and problem gambling neither among females (*r*_pb_ =  − 0.080, *N* = 571, *p* = 0.057) nor males (*r*_pb_ = − 0.054, *N* = 519, *p* = 0.217).

### Multivariable Associations

Each of the final logistic regression models for the overall sample (${\upchi }$_(df = 18)_ = 272.54, *p* < 0.001), female sub-sample (${\upchi }$_(df = 17)_ = 146.84, *p* < 0.001), and the male sub-sample (${\upchi }$_(df = 17)_ = 130.22, *p* < 0.001) was statistically significant, accounting for 75%, 76%, and 74% of the variance within the outcome variable in the overall, female, and male models respectively. Table 3 shows the results of the multivariable analysis of demographic and exposure variables associated with problem gambling in the previous 12 months, stratified by gender.

**Table 3:** Multivariable analysis of demographic and exposure variables associated with problem gambling in the previous 12 months, stratified by gender

	Model 1: Total sample (n = 1101)				Model 2: Female (n = 575)				Model 3: Male (n = 526)			
Variables in models	*β*	AOR	95% CI	*p* value	*β*	AOR	95% CI	*p *value	*β*	AOR	95% CI	*p *value
*Demographic variables*												
Gender	**0.64**	**1.89**	**1.40, 2.56**	**0.000**								
Age	**− 0.36**	**0.69**	**0.51, 0.93**	**0.015**	**− **0.24	0.78	0.51, 1.20	0.263	**− 0.49**	**0.61**	**0.40, 0.93**	**0.022**
*Grade*												
JHS 1	Reference				Reference				Reference			
JHS 2	0.20	1.22	0.87, 1.71	0.242	0.33	1.39	0.84, 2.31	0.190	0.14	1.15	0.72, 1.84	0.553
JHS 3	**− 0.71**	**0.49**	**0.28, 0.84**	**0.010**	**− 0.84**	**0.43**	**0.19, 0.96**	**0.041**	**− **0.58	0.55	0.26, 1.18	.128
*Living arrangement*												
Live with both parents	Reference				Reference				Reference			
Live with one parent	**− **0.10	0.90	0.62, 1.29	0.580	**− **0.41	0.65	0.38, 1.12	0.125	0.15	1.16	0.70, 1.93	0.557
Live with no parents	**− **0.28	0.76	0.48, 1.19	0.228	**− **0.68	0.50	0.25, 1.00	0.050	0.06	1.06	0.56, 2.00	0.844
Caretaker's employment status	**− **0.20	0.81	0.51, 1.29	0.384	0.24	1.27	0.57, 2.85	0.549	**− **0.42	0.65	0.36, 1.16	.150
Family structure	**0.35**	**1.41**	**1.03, 1.93**	**.031**	**0.49**	**1.64**	**1.02, 2.63**	**0.038**	0.25	1.29	0.83, 2.01	0.249
In romantic relationship	**0.42**	**1.51**	**1.09, 2.09**	**0.011**	0.13	1.14	0.71, 1.82	0.565	**0.69**	**1.99**	**1.25, 3.15**	**0.003**
*Personal factors*												
Religious participation	**− 0.10**	**0.90**	**0.82, 0.99**	**0.043**	**− **0.08	0.91	0.79, 1.05	0.232	**− **0.09	0.91	0.79, 1.04	0.189
Weekly alcohol use	**0.66**	**1.94**	**1.37, 2.73**	**0.000**	0.45	1.57	0.95, 2.58	0.076	**0.80**	**2.23**	**1.34, 3.70**	**0.002**
Truancy	0.27	1.31	0.82, 2.10	0.254	0.22	1.24	0.59, 2.61	0.555	0.29	1.33	0.69, 2.56	0.378
Schoolwork problems	0.25	1.28	0.93, 1.75	.118	0.18	1.19	0.75, 1.90	0.446	0.34	1.41	0.91, 2.20	0.122
*Social adversities*												
Bullying victimisation	**0.47**	**1.59**	**1.18, 2.15**	**0.002**	0.40	1.49	0.96, 2.31	0.071	**0.55**	**1.74**	**1.12, 2.68**	**0.012**
Breakup	**0.49**	**1.63**	**1.17, 2.25**	**0.003**	**0.70**	**2.01**	**1.24, 3.26**	**0.004**	0.32	1.38	0.86, 2.20	0.180
Sexual abuse victimisation	**1.09**	**2.99**	**2.17, 4.12**	**0.000**	**1.23**	**3.43**	**2.15, 5.48**	**0.000**	**1.03**	**2.81**	**1.75, 4.53**	**0.000**
Conflict with parents	0.22	1.24	0.88, 1.75	.214	0.10	1.10	0.67, 1.81	0.693	0.24	1.27	0.78, 2.08	0.331
Parental divorce	**0.36**	**1.43**	**1.02, 2.00**	**0.038**	**0.69**	**2.00**	**1.22, 3.26**	**0.005**	**− **0.00	0.99	0.61, 1.62	.989
Nagelkerke pseudo R^2^		0.306				0.322				0.301		
Cox and Snell R^2^		0.221				0.227				0.222		
Hosmer–Lemeshow GOF test (sig.)		6.23 (.621)			3.94 (.862)			11.77 (.162)				
Overall percentage correctly classified		75.0				76.0				74.4		

*β *beta value; *AOR *adjusted odds ratios; *CI *Confidence Interval; *GOF *goodness of fit; statistically significant results are in bold face

### Factors Associated with Problem Gambling in Overall Sample

As shown in Table 3, sexual abuse victimisation (AOR = 2.99; 95% CI = 2.17, 4.12), weekly alcohol use (AOR = 1.94; 95% CI = 1.37, 2.73), male gender (AOR = 1.89; 95% CI = 1.40, 2.56), breakup (AOR = 1.63; 95% CI = 1.17, 2.25), bullying victimisation (AOR = 1.59; 95% CI = 1.18, 2.15), being in a romantic relationship (AOR = 1.51; 95% CI = 1.09, 2.09), parental divorce (AOR = 1.43; 95% CI = 1.02, 2.00), and being in a polygamous family—father having more one wife (AOR = 1.41; 95% CI = 1.03, 1.93) were significantly associated with increased odds of problem gambling. However, religious participation (AOR = 0.90; 95% CI = 0.82, 0.99), being an older adolescent (AOR = 0.69; 95% CI = 0.51, 0.93) and being in JHS 3 (AOR = 0.49; 95% CI = 0.28, 0.84) were associated with reduced odds of problem gambling.

### Gender Difference and Commonality in Factors Associated with Problem Gambling

Among females, sexual abuse victimisation (AOR = 3.43; 95% CI = 2.15, 5.48), breakup (AOR = 2.01; 95% CI = 1.24, 3.26), parental divorce (AOR = 2.00; 95% CI = 1.22, 3.26), and being in a polygamous family (AOR = 1.64; 95% CI = 1.02, 2.63) were associated with increased odds of problem gambling; however, being in JHS 3 (AOR = 0.43; 95% CI = 0.19, 0.96) was associated with reduced odds of problem gambling–see Table 3.

Table 3 also shows that among male adolescents, whereas sexual abuse victimisation (AOR = 2.81; 95% CI = 1.75, 4.53), weekly alcohol use (AOR = 2.23; 95% CI = 1.34, 3.70), being in a romantic relationship (AOR = 1.99; 95% CI = 1.25, 3.15), and bullying victimisation (AOR = 1.74; 95% CI = 1.12, 2.68), were associated with increased odds of problem gambling, being an older adolescent (AOR = 0.61; 95% CI = 0.40, 0.93) was associated with reduced odds of problem gambling.

Thus, it is notable that sexual abuse victimisation emerged as the strongest and only factor associated with the increased odds of problem gambling across the overall sample (AOR = 2.99; 95% CI = 2.17, 4.12), among females (AOR = 3.43; 95% CI = 2.15, 5.48) and male adolescents (AOR = 2.81; 95% CI = 1.75, 4.53).

## Discussion

We have found in this study that overall, three in 10 in-school adolescents (representing, approximately, 3 in 10 females; 4 in 10 males) in rural Ghana reported problem gambling in the previous 12 months. Female adolescents who experienced family-related social adversities were likely to report problem gambling, while adolescent male problem gambling was likely to be associated with school-related factors and interpersonal factors outside the family. Regardless of gender, in-school rural adolescents who reported sexual abuse were about three times more likely to experience problem gambling, compared to those who did not report sexual abuse victimisation.

### Prevalence of Problem Gambling

Generally, the 12 month prevalence estimate of problem gambling found in this study (34.3%) is higher, relative to estimates from high-income countries, which range from 0.2% to 12.3% (Calado et al. 2017; Hardoon et al. 2004). However, as should be expected, the reported prevalence estimates of the current study are comparable to estimates from other countries within sub-Saharan Africa, including Nigeria (Aguocha et al. 2019) and Ethiopia (Abdi et al. 2015). Again, consistent with other studies from the subregion (Abdi et al. 2015; Aguocha et al. 2019; Glozah et al. 2019; Sharp et al. 2015). Beyond possible individual-level motivational factors, the higher prevalence estimates in current study could be attributable to the social acceptability of gambling for the purposes of financial gains and the relatively increased adolescent access to gambling centres or sources in rural Ghana (Department of Social Welfare 2018; Hayk and Sailer 2020). The Gaming Act of Ghana^4^ prohibits children from gambling and forbids operators of gambling centres and machines from allowing children to patronise. However, a recent report by the Department of Social Welfare in the Ayensuano rural district (where this study was conducted) suggests that child and adolescent gambling is now a public concern and thus recommends that, “Game center operators should be severely dealt with since adolescents patronize it… Chinese Jackpot games should be seized” by the authorities (p.6) (Department of Social Welfare 2018). Also, the most recent published evidence from rural Ghana suggests that Chinese slot machines are a bane of the life of rural-dwelling young people (Hayk and Sailer 2020). Given this contextual reality, we are not entirely surprised at the prevalence estimate of probable problem gambling in this study, even though it appears comparatively higher.

### Factors Associated with Problem Gambling

Generally, the correlates of problem gambling found in this study (sexual abuse victimisation, weekly alcohol use, male gender, breakup, bullying victimisation, being in a romantic relationship, parental divorce and being in a polygamous family) are consistent with evidence in the global literature (Derevensky et al. 2011; Floros 2018; Grande-Gosende et al. 2020; Hayer and Griffiths 2015; Lane et al. 2016; Molinaro et al. 2014; Shead et al. 2011). Specifically, this study shows that regardless of gender, participants who reported sexual abuse victimisation were about three times more likely to experience problem gambling. This finding is consistent with an important evidence in the area, that adolescent victims of sexual abuse are at elevated risk of problem gambling. It has been argued that adolescent victims of sexual abuse are more likely to take to gambling as a means of emotional coping, given that the problem solving capacities and coping skills are not fully formed during adolescence (Blaszczynski and Nower 2002; Felsher et al. 2010; Lane et al. 2016). Similarly, female adolescents who report a breakup and parental divorce are likely to gamble as a means of emotional coping and ‘escaping’ their psychological and social pains. In other words, adolescent problem gambling can be interpreted as a response to traumatic experiences (Derevensky et al. 2011; Felsher et al. 2010; Messerlian et al. 2005). A notable finding of this study is that females from polygamous families are more likely to report problem gambling. This evidence is to be expected, as young girls in polygamous families tend to be the most disadvantaged; there is evidence to suggest that compared to boys, girls in polygamous families experience more corporal punishment and lower academic performance (Al-Sharfi et al. 2016). Even so, considering that most polygamous families are poor, it is not readily clear from this study whether adolescent girls from polygamous families engage in gambling with the motive to make money to support themselves or as a means of coping or escaping the social adversities in larger families. Perhaps qualitative studies may prove more informative in exploring the firsthand accounts and individualised meanings of female adolescents from polygamous families.

The findings of this study show weekly alcohol use as a strong positive correlate of problem gambling among male adolescents. Whereas this evidence is consistent with reports from both high-income and low- and middle-income contexts (Aguocha et al. 2019; Shead et al. 2010), the explanation of the exact risk and causal relationships between alcohol use and gambling is not straightforward, even though previous evidence has linked alcohol use to impulsivity and health risk-taking behaviours among adolescents (Inchley et al. 2018). Problem gambling could be related to the odds of alcohol use, as problem gamblers tend to experience problem solving and emotion regulation difficulties (Jauregui et al. 2016), and alcohol use could be a factor for problem gambling, but each of these factors could also be outcomes influenced separately by other factors (Barnes et al. 1999; Cronce and Corbin 2011; Huggett et al. 2019; Jauregui et al. 2016; Sagoe et al. 2017). A recent twin study suggests that shared genetic and (or) risk factors in the environment influence the association between frequent alcohol use and increased gambling (Huggett et al. 2019). Furthermore, being in a romantic relationship was associated with increased odds of problem gambling among male adolescents. Typically, in Ghana, adolescent males in heterosexual romantic relationships assume the role of providing financial support to their girlfriends; girlfriends are financially dependent on their boyfriends (Ampofo 2001). This implies that, perhaps, for an unemployed school-going male adolescent, gambling could represent a source of earning some money to provide financial support to their girlfriend. Future studies could explore further the key motivational factors undergirding problem gambling among in-school male adolescents who are in romantic relationships.

Three factors, even though with relatively lower effect sizes, showed associations with reduced odds of problem gambling: religious participation/attendance, older age, and being in a higher school grade. Older adolescents in Junior High School grade 3, who participated in religious activities or attended religious services frequently were less likely to report problem gambling. Whereas this evidence supports previous findings in the area (Dowling et al. 2017; Floros 2018), our results do not demonstrate whether these behavioral factors are key ‘protective’ variables against the onset of problem gambling.

### Strengths and Limitations

This study is partly in response to the call for expansive studies on problem gambling among young people in countries within sub-Saharan Africa (Ssewanyana and Bitanihirwe 2018). This study represents the first attempt from a sub-Saharan Africa country at providing a systematic evidence on the prevalence of problem gambling and some personal and interpersonal correlates among rural in-school adolescents. Nonetheless, some plausible limitations of the study are noteworthy. The multiple responses of the DSM-IV-MR-J scale are collapsed when computing the scores; this could lead to insufficient evidence in support of the classification accuracy of the scale (Derevensky and Gupta 2005b; Edgren et al. 2016; Stinchfield 2011). Relatedly, as adolescent gambling represents a relatively new area of research (Blinn-Pike et al. 2010), most of the current screening measures (including DSM-IV-MR-J scale) of gambling in young people lack sufficient construct validity (Blinn-Pike et al., 2010; Derevensky and Gupta 2000, 2005b). It has been suggested, for example, that the DSM-IV-MR-J scale results in overestimates of the prevalence of probable problem gambling among young people (Derevensky et al. 2003). However, while we acknowledge that our reported prevalence estimates may be inflated, we also agree with the argument by leading youth gambling researchers that when the number of adolescents experiencing gambling challenges is as high as reported in research, more adolescents are likely to present for treatment (Derevensky et al. 2003).

Furthermore, there is evidence to suggest that studies on gambling among young people are susceptible to social desirability bias (i.e., self-deceptive enhancement effects, and impression management effects) (Kuentzel et al. 2008). However, in our study we believe that this bias might be low, as participants were allowed enough privacy in responding to the survey: we used an anonymous self-report questionnaire, participants sat as far apart from one another as possible while answering the questionnaire, and teachers were kept in the background during the survey. It is also notable that causal interpretation of our findings is not possible, given the cross-sectional design used (Woodward 2014). Lastly, our study failed to include absentee students on the day of the study and out-of-school rural-dwelling adolescents. This implies that whereas our findings may be generalisable across non-clinical sample of in-school adolescents in rural eastern Ghana (and other rural contexts in sub-Saharan Africa), they may not necessarily apply to their peers who are out of school. Besides not including an urban adolescent sample to facilitate a comparative ecological analysis in this study, our study also failed to consider some variables which could present as covariates of probable problem gambling among adolescents. For example, personality factors, anxiety, depression, social acceptability, family/parental permissiveness of adolescent gambling, access and proximity to gambling points and sources, among personal, family and locality factors (Derevensky et al. 2011; Derevensky and Gupta 2005a).

### Implications and Recommendations

Clearly, more research is needed to expand the evidence base on adolescent (problem) gambling in rural Ghana, as the findings of this study come from only one rural district. Specifically, cross-sectional surveys and interview-based studies that identify the motivations of rural adolescents for gambling are needed, but also longitudinal studies using robust designs are needed to understand the risk and protective factors, and patterns and changes in gambling behaviour across adolescence through early adulthood. Potentially, future studies of this nature would be more useful in informing intervention, prevention and health promotion programmes. As recommended by recent systematic reviews (Floros 2018; Keen et al. 2017; Ladouceur et al. 2013), the position of this study is that any intervention and prevention efforts and treatment models should be theory-driven and evidence-based. Drawing on the evidence of this study, school-based psychoeducation and harm reduction prevention programmes could be instituted in schools within rural Ghana, following universal, selective, and indicative prevention frameworks. This approach has been found to be potentially useful in the treatment and prevention of gambling among young people (Dickson et al. 2004; Floros 2018; Ladouceur et al. 2003). The evidence in this study also underscores the need for the government of Ghana (through the Ghana Police Service and the Department of Social Welfare) to enforce strictly the abstinence law on underage gambling, but also pursue and implement the relevant educational and social policies aimed at enhancing supportive school climate, mitigating familial adversities, and protecting adolescents (particularly, young girls) from abuses.

## Conclusion

Relative to the prevalence of gambling among adolescents in urban contexts in other countries in sub-Saharan Africa, the estimates of problem gambling among in-school rural adolescents in Ghana are higher. Although further studies are needed to understand the nuances of the behaviour, the evidence of this study underscores the need for general and targeted health promotion, intervention and prevention efforts to mitigate the family, school, and interpersonal social adversities associated with adolescent problem gambling in rural Ghana.

## Electronic Supplementary Material

Below is the link to the electronic supplementary material.

Supplementary material 1 (DOCX 15 kb)

## Data Availability

The datasets used and/or analysed during the current study are available from the corresponding author on reasonable request.

## Abbreviations

DSM-IV-MR-J: Diagnostic and Statistical Manual of Mental Health Fourth Edition Adapted for Juveniles
DUREL: Duke University Religion Index
JHS: Junior High School
WHO: World Health Organization

## Footnotes

1 In order to contextualise our study specifically within Ghana and generally within Africa, we conducted a systematic search for literature from the sub-region. We searched African Journals OnLine, African Index Medicus, CINAHL, EMBASE, MEDLINE, and PsycINFO, up to May 2019, and updated to February 2020, using keywords [e.g., (“problem gambling” OR gambl* OR “betting”) AND (Adolescen* OR students OR “rural adolescents” OR child* OR teen* OR “young people”)]. We did not apply any language or date limits. For our geographic search filter, we included names of African countries in both English and languages relevant to the countries. We search the references of the identified key papers and used Google to look for relevant forward citations of key papers.

2 Based on the definition by the WHO (2009), we used the term adolescents to denote individuals between 10 and 19 years. [WHO. (2009). *Strengthening the health sector response to adolescent health and development*. Geneva, Switzerland: WHO.].

3 In Ghana, Junior High Schools are targeted at young people aged 10–13 years, but due to delayed school enrolment in rural communities, typically, older and late adolescents aged 14–19 years are also predominantly found at this level of basic education in rural Ghana.

4 The Gaming Act 721 (2006) of Ghana, stipulates in: Section 56(2): A person who permits a child to use a gambling machine or to enter a place in which a gambling machine is used commits an offence and is liable on summary conviction to a fine of not more than two hundred and fifty penalty units or a term of imprisonment of not more than twelve months or both. Section 56 (3): A child who uses a gambling machine, or enters a place in which a gambling machine is used, commits an offence and is liable on summary conviction by a juvenile court to a fine of not more than one hundred penalty units or a custodial sentence that the court determines.
