# Supplementary material for: Adolescent Problem Gambling in Rural Ghana: Prevalence and Gender Differentiation
Source: J Gambl Stud. 2020 Nov 11;37(1):83–105. doi: 10.1007/s10899-020-09987-6 (PMC7882566; doi:10.1007/s10899-020-09987-6)
Supplement: Supplementary file 1 — Supplementary material 1 (DOCX 15 kb) [file 10899_2020_9987_MOESM1_ESM.docx]

**Supplementary material**

eTable 1. Coding of variables

| Variable | Survey question | Response coding |
| --- | --- | --- |
| Demographic variables: |  |  |
| Gender | Are you a girl or a boy? | 0 = Girl  1 = Boy |
| Age | How old are you? Please write your age. | 10 – 19 years (continuous), but re-coded  0 = Younger adolescents (10 – 15 years)  1 = Older adolescents (16 – 19 years) |
| Grade | In which form are you? | 0 = JHS1  1 = JHS 2  2 = JHS 3 |
| Living arrangement | Whom do you live with? I live with | 0 = Both parents  1 = One parent  2 = No parents |
| Caretaker's employment status | What is the employment status of the person who is most responsible for taking care of you and providing your needs? | 0 = Unemployed  1 = Employed |
| Family structure | How many wives does your father have? | 0 = 1 wife  1 = more than 1 wife |
| In romantic relationship | Do you have a boyfriend or a girlfriend? | 0 = No  1 = Yes |
| Personal factors: |  |  |
| Religious participation | How often do you attend church or other religious meetings? | (coded continuously):  1 = Never  2 = Once a year or less  3 = A few times a year  4 = A few times a month  5 = Once a week  6 = More than once/week |
| Weekly alcohol use | In a typical week, how many times do you have at least on alcoholic drink? | 0 = Never  1 = 1 or more drinks |
| Truancy | During the past 12 months, how many days did you miss classes or school without permission? | 0 = 0 – 5 days  1 = More than 5 days |
| Schoolwork problems | Have you had difficulties with your studies at school during the previous 12 months? | 0 = No  1 = Yes |
| Social adversities: |  |  |
| Bullying victimisation | During the past 12 months, how many days were you bullied? | 0 = Never  1 = 1 or more times |
| Breakup | Have you had a breakup with your boyfriend or girlfriend during the past 12 months? | 0 = No  1 = Yes |
| Sexual abuse victimisation | Has anyone forced you (i.e. physically or verbally) to engage in sexual activities against your will? | 0 = No  1 = Yes |
| Conflict with parents | Have you had any serious arguments or fights with one or both of your parents, during the past 12 months? | 0 = No  1 = Yes |
| Parental divorce | During the past 12 months, have your parents divorced? | 0 = No  1 = Yes |
